# Environment-Driven Coherent Population Transfer Governs the Ultrafast Photophysics of Tryptophan

**DOI:** 10.1021/jacs.2c04565

**Published:** 2022-07-07

**Authors:** Vishal Kumar Jaiswal, Piotr Kabaciński, Barbara E. Nogueira de Faria, Marziogiuseppe Gentile, Ana Maria de Paula, Rocio Borrego-Varillas, Artur Nenov, Irene Conti, Giulio Cerullo, Marco Garavelli

**Affiliations:** † Dipartimento di Chimica industriale “Toso Montanari”, Università di Bologna, Viale del Risorgimento 4, 40136 Bologna, Italy; ‡ Dipartimento di Fisica, Politecnico di Milano, Piazza Leonardo da Vinci 32, 20133 Milano, Italy; § Departamento de Física, Universidade Federal de Minas Gerais, 31270-901 Belo Horizonte-MG, Brazil; ∥ Istituto di Fotonica e Nanotecnologie, CNR-IFN, Piazza Leonardo da Vinci 32, 20133 Milano, Italy

## Abstract

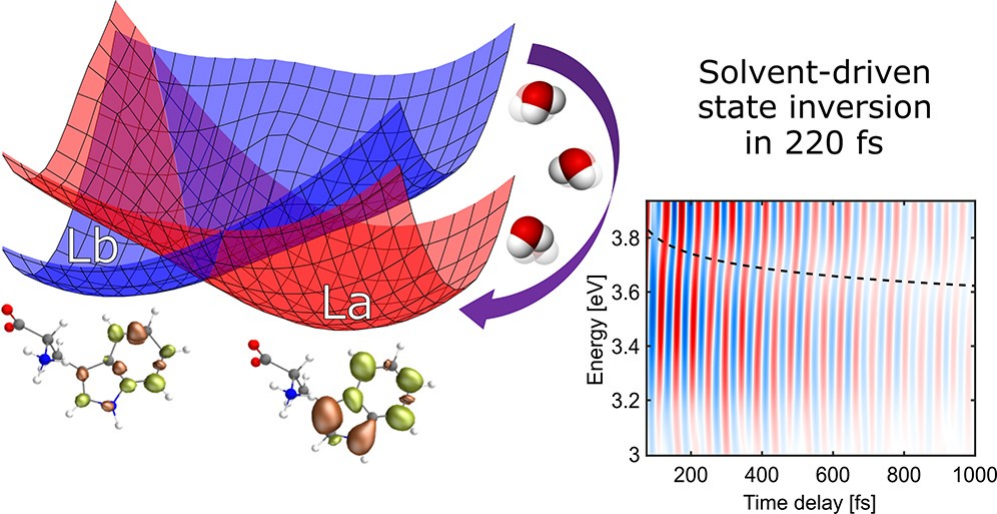

By combining UV transient absorption spectroscopy with sub-30-fs temporal resolution and CASPT2/MM calculations, we present a complete description of the primary photoinduced processes in solvated tryptophan. Our results shed new light on the role of the solvent in the relaxation dynamics of tryptophan. We unveil two consecutive coherent population transfer events involving the lowest two singlet excited states: a sub-50-fs nonadiabatic L_a_ → L_b_ transfer through a conical intersection and a subsequent 220 fs reverse L_b_ → L_a_ transfer due to solvent-assisted adiabatic stabilization of the L_a_ state. Vibrational fingerprints in the transient absorption spectra provide compelling evidence of a vibronic coherence established between the two excited states from the earliest times after photoexcitation and lasting until the back-transfer to L_a_ is complete. The demonstration of response to the environment as a driver of coherent population dynamics among the excited states of tryptophan closes the long debate on its solvent-assisted relaxation mechanisms and extends its application as a local probe of protein dynamics to the ultrafast time scales.

## Introduction

The photophysics of the aromatic amino acid tryptophan (Trp) is a subject of active investigation because of its intrinsic importance in biochemistry and its applications as a local probe to follow protein structural dynamics, thanks to its intense fluorescence.^1−6^ The ultrafast dynamics in Trp triggered by UV light absorption have been the subject of numerous experimental and theoretical studies; however, the early stages of excited state (ES) relaxation have remained unexplored and the role of the environment in determining its decay mechanisms is highly debated.^7−22^

**Figure 1:**
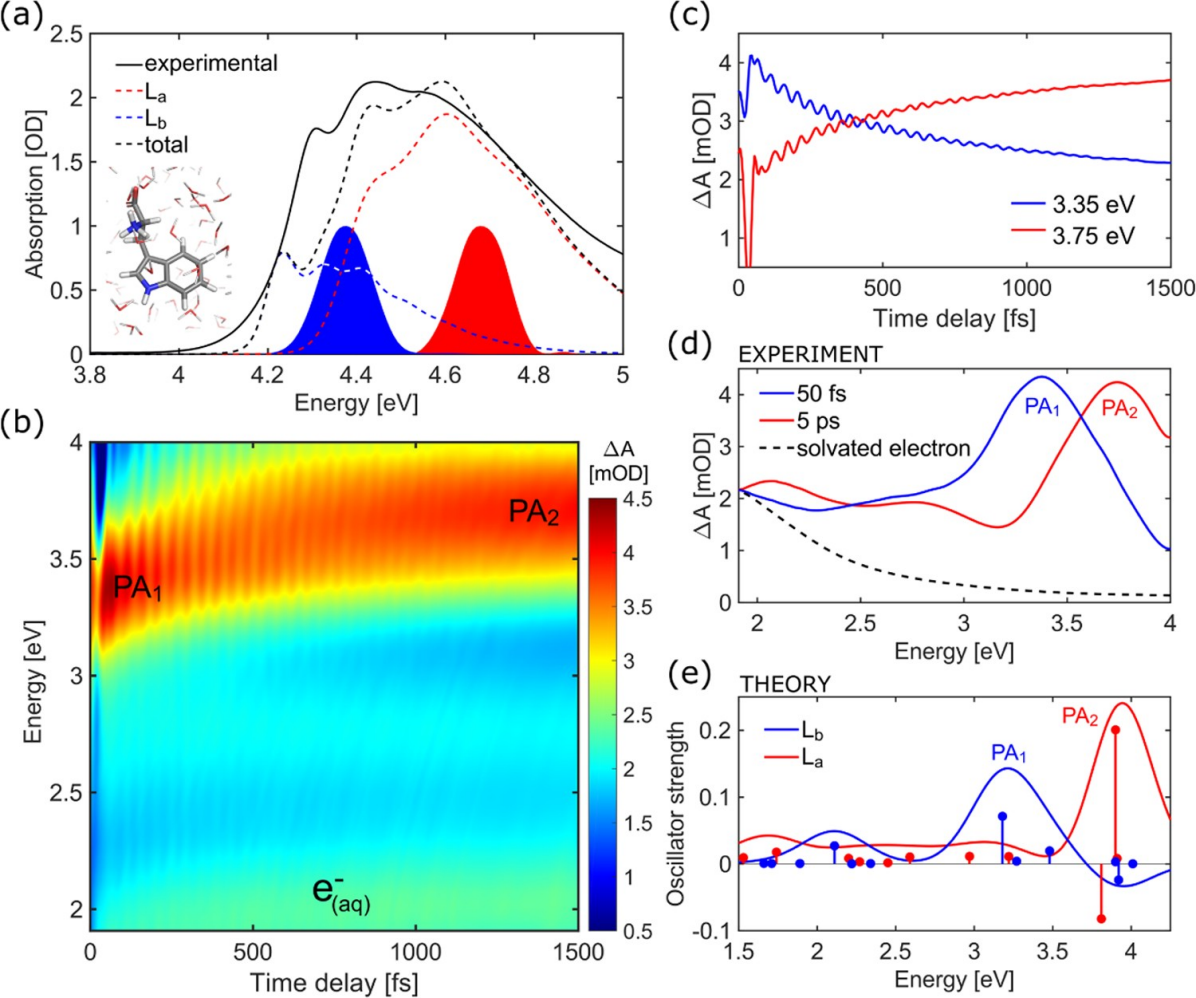
(a) Experimental steady-state absorption spectrum of Trp in a phosphate buffer solution at pH 7.4 compared to the theoretical spectrum computed at XMS-CASPT2 level with an expanded active space of (0|10,9|2,4) in a displaced harmonic oscillator formalism. The two pump pulse spectra are depicted with blue and red filled curves. Inset shows the molecular structure of Trp. (b) Experimental transient absorption (Δ*A*) map following excitation at 4.37 eV. (c) Δ*A* time traces at selected probe photon energies showing simultaneous decay of PA_1_ and rise of PA_2_. (d) Experimental TA spectra at 50 fs and 5 ps after photoexcitation with a 4.37 eV pulse. The PA spectrum of the solvated electrons expected to contribute to the visible region [33] is also shown as a dashed line. (e) Theoretical TA spectra (positive for PA, negative for SE) from the L_b_ and L_a_ states computed at their respective excited-state minima.

Trp exhibits two close-lying bright ππ* ES with overlapping absorption bands, a nonpolar L_b_ and a polar L_a_ state (Figure 1a), which are simultaneously populated upon irradiation with UV light (4.2–4.8 eV). The different electronic nature of the two states implies a different sensitivity to the local environment, evoking complex nonadiabatic internal conversion (IC) dynamics accompanied by state-order inversion in polar solvents. L_a_ lies vertically above L_b_ in the Franck–Condon (FC) geometry, but it is the fluorescent state in polar solvents, collecting the ES population.^23,24^

For this reason, the ultrafast spectral evolution observed in polar solvents has been attributed to the L_b_ → L_a_ IC process.^7,8,15−17^ The time scale of this process has been a subject of debate in the literature. Ruggiero et al.^15^ attributed to it a time constant of 1.6 ps, while Chergui et al.^16,25^ assigned a sub-100-fs time constant, followed by a biexponential solvent relaxation with time scales of 160 ± 40 fs and 1.02 ± 0.12 ps. Shen et al.^7^ and Sharma et al.^8,20^ also attributed the picosecond time constant to solvent relaxation dynamics, thus implying that the IC is a sub-ps event.

Considering the ability of broadband ultrashort laser pulses to coherently excite a superposition of vibronic states, it is illuminating to regard the photophysics of Trp through a quantum wave packet-based formalism. Yang et al.^17^ analyzed their time-resolved fluorescence data with this approach, considering the state prepared after 290 nm (4.27 eV) excitation as a superposition of L_a_ and L_b_ states, and postulated that the decay of the superposition as well as the L_b_ → L_a_ IC occurs on a sub-100-fs time scale.

There is a distinct lack of theoretical studies on the electronic properties of solvated Trp. However, work done on indole, the chromophore of Trp, can shed light on the photophysics triggered by UV photoexcitation.^18,26−31^ Giussani et al.^18^ mapped out the minimum energy paths (MEPs) in the gas phase at the CASSCF//CASPT2 level of theory, thereby demonstrating that, in the absence of a polar solvent, L_b_ is energetically more stable with respect to L_a_. They reported two conical intersections (CIs) between L_b_ and L_a_, hinting at a potential L_a_ → L_b_ population transfer immediately after photoexcitation that had remained elusive in time-resolved experiments. The existence of a strong vibronic coupling required for an efficient IC was demonstrated by Brand et al.^26,27^ Relying on nonadiabatic dynamics in explicit solvent using time-dependent density functional theory, Wohlgemuth et al.^19^ obtained a time constant of 45 fs for the L_a_ → L_b_ IC, along with a minor repopulation of the ground state (GS) through a πσ* state accessed from L_a_.^10,32^ These studies suggest the intriguing idea that the early excited-state dynamics in Trp are characterized by a sub-ps, CI-assisted, solvent-sensitive, and back-and-forth population transfer between L_a_ and L_b_.

Here, we combine transient absorption (TA) spectroscopy with sub-30-fs temporal resolution and theoretical computations at CASPT2 level,^33^ incorporating solvent effects within a hybrid quantum mechanics (QM)/molecular mechanics (MM) setup, to unveil the early stages of excited-state relaxation in solvated Trp. We provide compelling experimental evidence of the hypothesized sub-50-fs initial population transfer from L_a_ to L_b_, which is followed by a solvent-driven repopulation of the L_a_ state with a 220 fs time constant. We demonstrate that solvent reorganization dynamics dictate the electronic state order and drive the direction of the population transfer. Finally, we show that a vibronic coherence established upon photoexcitation and lasting several picoseconds is a signature of the L_a_/L_b_ coupling and facilitates the coherent population transfer dynamics.

## Results and Discussion

We investigate the ultrafast dynamics of Trp following photoexcitation at 4.70 eV (264 nm) and 4.37 eV (284 nm). As seen in Figure 1a, the two pump pulses (filled curves) are tuned to excite predominantly either the L_a_ or the L_b_ electronic state to follow the decay pathways associated with each state. Figure 1b reports the TA map, as a function of probe photon energy and delay, for the 4.37 eV pump (L_b_ centered), while Figure S1 in the Supporting Information (SI) compares it to the TA map for the 4.70 eV pump. The two maps show remarkable similarities, suggesting that the photoinduced dynamics are independent of the nature of the initially populated state. Positive differential absorption (Δ*A*) signals are observed over the entire probe photon energy window, indicating the presence of intense photoinduced absorption (PA) bands, which cover the stimulated emission (SE) from the ES, expected in the 3.75–4 eV range based on QM/MM SS-CASPT2 estimations from optimized ES minima (Table S1). In particular, the spectral evolution above 3 eV is characterized by the disappearance of a strong PA band at 3.37 eV (PA_1_) and the simultaneous appearance of a band at 3.76 eV (PA_2_) on the picosecond time scale (see Figure 1c for Δ*A* dynamics at selected probe photon energies), with an isosbestic point at 3.54 eV (Figure 1d). Global analysis of the experimental TA spectra reveals two time constants of 220 fs and 1.1 ps that describe this process (see Figure S2b in the Supporting Information).

Figure 1d,e shows a comparison of the experimental TA spectra at 50 fs and 5 ps time delays with the calculated transitions to a manifold of higher-lying ES starting from either L_b_ or L_a_. This comparison allows us to identify the PA_1_ and PA_2_ bands as fingerprints of L_b_ and L_a_, respectively, and shows that L_b_ is populated at early times independent of the pump photon energy, whereas the population resides in L_a_ at later times. Furthermore, less intense PA signals are observed below 3 eV, where L_b_ and L_a_ exhibit weaker absorption features, as documented in Figure 1e. In our experimental data, this region is obscured by the PA from solvated electrons formed as a minor byproduct due to the photoionization of the indole chromophore^34^ (dashed line in Figure 1d). At higher pump energies (4.70 eV), a nonradiative pathway from L_a_ to the πσ* state opens up an additional channel for the production of solvated electrons^28,35^ (see Supporting Information, Figure S1c). The Trp^+^ cation is expected to contribute to the PA of around 2 and 3.5 eV,^36^ in agreement with theoretical transient spectra (see Supporting Information, Figure S3).

The appearance of TA signals characteristic of the L_b_ state at very early times (50 fs) even after exciting predominantly the L_a_ state (4.70 eV pump) is strong evidence of an ultrafast, CI-mediated L_a_ → L_b_ IC process occurring at a sub-50-fs time scale, whose direct observation is obscured by the coherent artifact in the TA spectra during a pump-probe temporal overlap and suggests that L_b_ initially lies at lower energy with respect to L_a_. The observation of a TA signal characteristic of the L_a_ state at 5 ps delay implies an inversion of the order of these states on the picosecond time scale and a back and forth L_a_ ↔ L_b_ population transfer. We show that these peculiar dynamics can be explained by the vibronic coherence established between the two states and the dynamic solvent response to the substantially different electronic nature of the L_b_ and L_a_ states.

**Figure 2:**
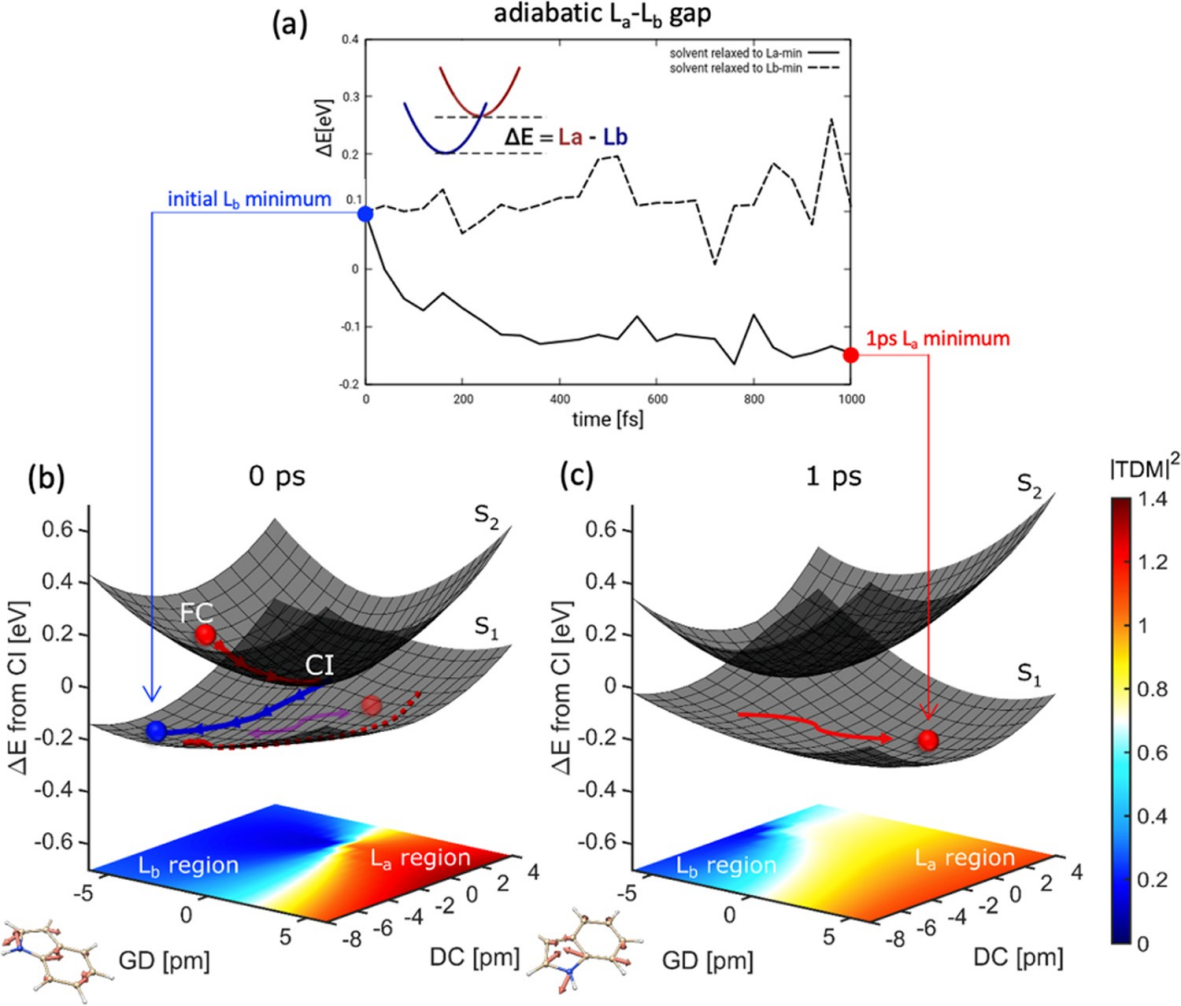
(a) Time evolution of adiabatic gap (Δ*E* = L_a_ – L_b_) between L_a_ and L_b_ states at their respective minima in L_a_-relaxed solvent (solid line) versus L_b_-relaxed solvent (dashed line). (b) Branching space with solvent relaxed to ground-state electron density, representative of early times after photoexcitation, and (c) after 1 ps of nonequilibrium solvent dynamics around the L_a_ state. The molecular deformations associated with the derivative coupling (DC) and gradient-difference (GD) vectors are also depicted with arrows. The *xy*-plane shows a heat map of the transition dipole moment (|TDM|^2^) from the GS to the lower adiabatic surface (S_1_). Colors allow to characterize the nature of the S_1_ surface as L_a_ (red), L_b_ (blue), or mixed (cyan/white/yellow). In (b), red and blue lines denote the projection of MEP from the FC point to the CI and from the CI to the L_b_ minimum, respectively. The dashed red line depicts the projection of an unconstrained optimization from the L_a_ region, which smoothly leads to the L_b_ region on the lower surface. The tendency of a hot wave packet on the S_1_ surface to explore coherently L_a_ and L_b_ regions is shown schematically by a double-headed magenta arrow. In (c), a red arrow schematically depicts the stabilization of the L_a_ region on the S_1_ surface, promoted by the environment (solvent) reorganization, leading to a transfer of population from the L_b_ region.

Our calculations shed light on the charge localization of the L_a_ and L_b_ states and allow us to assign the initial global minimum before solvent relaxation. As can be seen from the difference in charge distribution with respect to the GS (Figure S4b), L_a_ is characterized by a significantly higher permanent dipole moment due to the intramolecular (5 → 6-membered ring) charge transfer nature of the electronic transition (Figure S4a). This contrasts with the L_b_ state, which is characterized by the delocalization of partial positive and negative charges resulting in a dipole moment with a magnitude similar to that of the GS. Then, when the solvent is relaxed to the GS electronic density, which is the case before photoexcitation, the minimum of the L_b_ state is more stable by only 0.1 eV than that of the L_a_ state (Figure 2a,b, values in Table S2), thus favoring an initial population transfer to the L_b_ state. In contrast to previous studies,^25,37^ we find the L_b_ state to be the initial global minimum in an aqueous environment instead of the more polar L_a_ state. However, the larger dipole moment of L_a_ is expected to induce a significant dynamic response (relaxation) of a polar solvent such as water. This solvent relaxation would thus cause the stabilization of L_a_ with respect to L_b_, which is initially lower in energy and less sensitive to the solvent reorganization, eventually leading to the inversion of the state ordering (see Figure 2a). In the following, we discuss in detail this coupled solute–solvent dynamics, which we can follow experimentally thanks to the high temporal resolution of our ultrafast TA setup.

We begin by focusing on the electronic structure of Trp immediately after the interaction with the pump pulse. As the solvent is in equilibrium with the GS electronic density, L_a_ is above L_b_ (ca. 0.3 eV vertical energy difference) at the FC point. The region of the potential energy surface (PES) relevant for the nonadiabatic dynamics at early times (i.e., before solvent relaxation kicks in) is conveniently displayed by means of the branching space around the L_b_/L_a_ CI (Figure 2b). The branching plane is defined by a pair of vectors termed gradient difference (GD) and derivative coupling (DC), which lift the degeneracy between the electronic surfaces, thereby giving rise to the characteristic double-cone topology of the PES around the CI (further details in the Experimental and Computational Methods section and in Section 11 of the Supporting Information). To aid the discussion, the electronic character of the lower adiabatic surface (termed S_1_) is depicted through the magnitude of the transition dipole moment from the GS, clearly demarcating regions of L_a_ (red) and L_b_ (blue) character, as well as regions of strong wavefunction mixing (cyan/white/yellow).

Vertical excitation from the FC region to the L_a_ state places the system on the upper adiabatic surface denoted as S_2_ (the L_a_ band being more intense than L_b_, Figure 1a). The ultrafast sub-50-fs population of L_b_ after excitation of the L_a_ state is demonstrated through the possible reaction paths taken by the photoexcited system. A minimum energy path (MEP) initiated on the S_2_ surface from the FC geometry (i.e., in the L_a_ state) reaches the CI in a barrierless fashion (red line). This motion predominantly occurs along the GD vector, which preserves the diabatic nature of the states and allows for minimal mixing. If we follow the direction of the S_2_ MEP (solid red line in Figure 2b) on the S_1_ surface, i.e., the trajectory of a momentum-conserving wave packet across the CI, we encounter a region of predominant L_a_ character (transparent red ball, Figure 2b), indicating that the CI crossing along the imaginary continuation of the MEP is to a large degree diabatic, i.e., character preserving. The L_a_ region on the lower S_1_ surface does not display a local minimum that could lead to population trapping. Instead, the branching space topography of the S_1_ surface provides a smooth relaxation pathway to the energetically more stable L_b_ region. This indicates that the L_a_/L_b_ vibronic coupling around the CI would allow for the relaxation to proceed on the S_1_ surface, circumventing the CI, and traversing a region of strong mixing to reach the energetically more stable L_b_ region. In support of this description, a full-dimensional geometry optimization initiated in the L_a_ region on S_1_ ends up in the L_b_ region (projection on the branching plane represented through a dashed red line in Figure 2b). An MEP from the CI region on the lower adiabatic S_1_ surface leads to a possible direct path to the L_b_ minimum (blue line in Figure 2b).

The branching plane vectors are shown along the axis of Figure 2b and comprise mostly high-frequency C–C and C–N stretching modes, which have periods shorter than 50 fs. A stretching mode of 1588 cm^–1^ leads to an inversion of the two states (Figure S6a). As shown in Figure 2b, the passage to the conical intersection from the Franck–Condon geometry can take place in 1/4 of a period of molecular vibration and therefore can occur in as short as 5 fs after the interaction with the pump. After the CI these, high-frequency modes lead to L_a_ and L_b_ regions on the lower adiabatic surface depicted by the two balls on the lower S_1_ surface in Figure 2b. The passage from the unstable L_a_ region to the L_b_ region on the S_1_ surface involves movement along the mixing modes exemplified in Figure S6b. Thus, within a single period of vibration of these high-frequency modes, all of the reaction paths can lead to the global L_b_ minimum from the Franck–Condon geometry. This explains why L_b_ fingerprints dominate the experimental TA spectrum already at early times even if pumped at 4.70 eV (Figure S1), where absorption from L_b_ is negligible. Taken together, the high-resolution TA data and the computed reaction pathways strongly suggest a sub-50-fs L_a_ → L_b_ IC process, in contrast to previous studies.^25^

The S_1_ PES is rather flat, with the L_b_ minimum region being only 0.1 eV more stable than the L_a_ region (see Figure 2b), allowing the “hot” wave packet arriving from S_2_ to spread and coherently explore regions of both L_b_ and L_a_ characters. This is essential as it allows fractions of the wave packet in the L_a_ region to polarize the environment, thereby inducing large-scale solvent reorganization leading to electrostatic relaxation of the system.^38,39^ To study the coupled solute–solvent dynamics, we modeled the response of the solvent to the electronic structure of either L_b_ or L_a_ by means of classical nonequilibrium dynamics. The Trp atomic charges were fitted to the electron density of the corresponding electronic state and the effect of the solvent reorganization on the relative stability of the L_a_/L_b_ ES minima of Trp was addressed by tracking the adiabatic L_a_–L_b_ energy gap during the dynamics (Figure 2a and further details in the Experimental and Computational Methods section).

Solvent relaxation around the L_b_ electron density preserves the state ordering (dashed line in Figure 2a), i.e., the L_b_ region remains more stable than the L_a_ region on the S_1_ surface throughout the dynamics. This renders improbable the previously proposed^17^ mechanism of L_b_ → L_a_ IC from a pure population in L_b_. In contrast, solvent relaxation around the L_a_ electron density reveals that inversion of the state ordering happens within 100 fs (solid line in Figure 2a) and the adiabatic stabilization of L_a_ continues on the picosecond time scale. This results in a drastic change in the PES topography, as demonstrated by the branching plane in a solvent environment after 1 ps of solvent reorganization dynamics (Figure 2c). As expected, the L_a_ region becomes more stable, which allows it to collect the S_1_ population through a back-transfer from L_b_. This adiabatic population transfer, in contrast to the sub-50-fs CI-mediated L_a_ → L_b_ IC process, is observed experimentally as the decay of the L_b_ (PA_1_) and simultaneous rise of L_a_ (PA_2_) fingerprint signals with a time constant of 220 fs (see fitted TA spectra in Figure S2 and calculated PA signals in Figure 1e).

Considering the pivotal role played by PA_1_ and PA_2_ in identifying the L_b_ and L_a_ states during the photoinduced dynamics, it is of paramount importance to address the effect of the coupled solute–solvent dynamics on the spectral fingerprints of those states. To this end, we compute the transient signals of the L_a_ state at different times between 80 fs and 5 ps along the solvent reorganization dynamics. The spectra (see Supporting Information, Figure S7) reveal that the relaxation of the solvent around the L_a_ electron density does not lead to any notable spectral shift in the PA signals. Thus, in contrast to the previous works,^8^ we can discard the interpretation that the disappearance of PA_1_ and the simultaneous appearance of PA_2_ are a consequence of a solvent-induced blue-shift of the PA signature of the L_a_ state.

**Figure 3:**
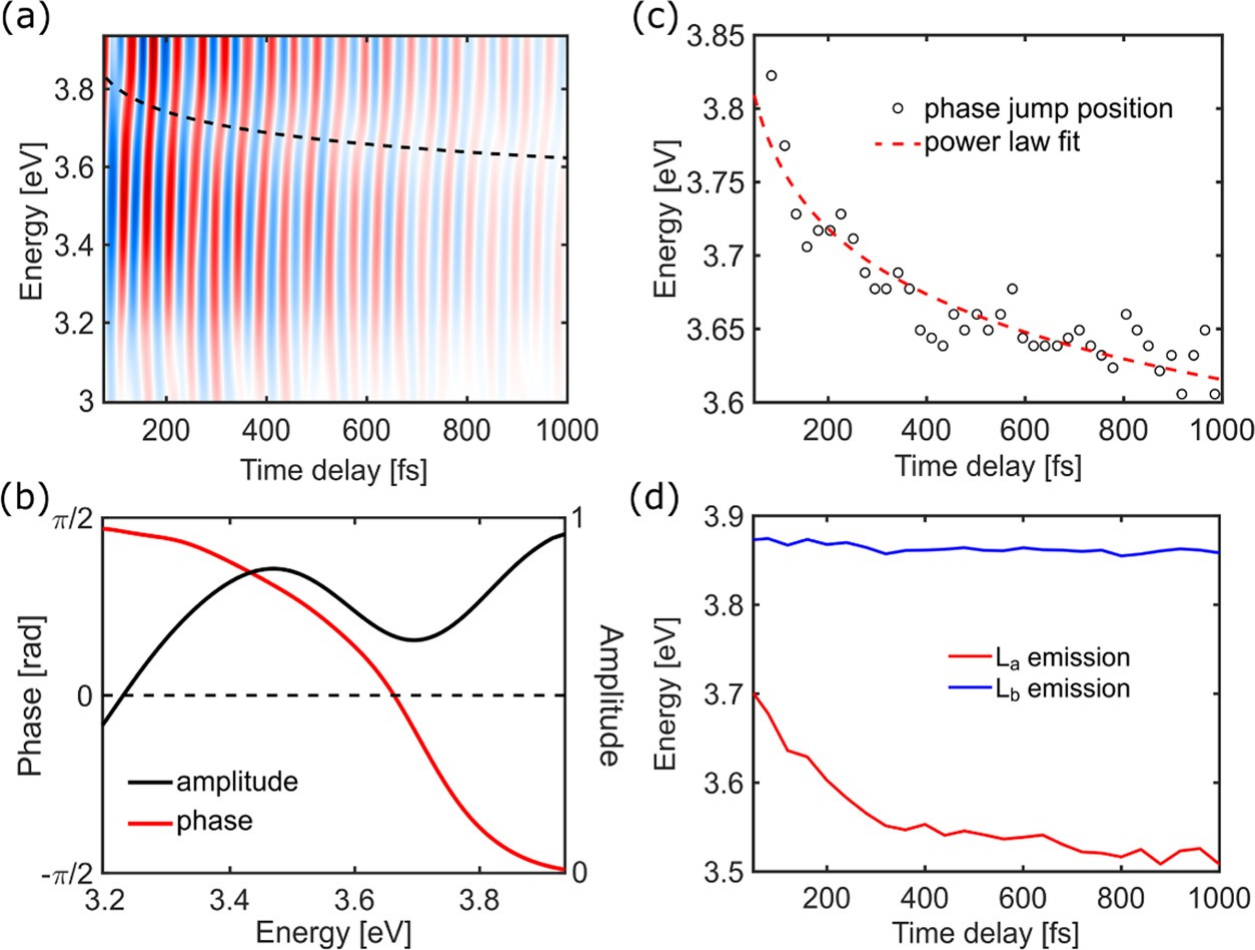
(a) Map of the oscillatory component of the TA map reported in Figure 1b, with the location of the phase jump tracked with the dashed line from the fit in panel (c). (b) Amplitude and phase of the FT of the oscillations from panel (a) for the 720 cm^–1^ frequency, showing a π-phase jump across the peaks that we associate with the central wavelength of the SE (here the node position is the average over the whole oscillatory trace). (c) Experimental phase jump positions read out from the map in panel (a), showing a continuous red-shift of the SE signal. (d) Emission energies computed at the SS-CASPT2 level along nonequilibrium relaxation of the solvent around the respective L_a_ and L_b_ ES minima.

Thanks to the high temporal resolution of our setup, which exceeds that of previous experiments on Trp by 1 order of magnitude,^8^ we observe coherent oscillations in the TA dynamics (Figure 1c), which encode the molecular vibrations active during the photoinduced processes. Figure 3a shows a map of the oscillatory component of the TA signal, following subtraction of the slow dynamics, as a function of probe photon energy and time. A Fourier transform (FT) of the map (Figure S8a) reveals a 720 cm^–1^ mode dominating the entire range of probe photon energies. Such an oscillation is a signature of a vibrational wave packet formed either in the ES or in the GS PES.^40^

The photon energy dependence of the amplitude and phase of the oscillations can be used to identify the state in which the wave packet is oscillating. The passage of the wave packet through the minimum of the PES results in a node in the FT amplitude, accompanied by a π-phase jump across the probe photon energy corresponding to the minimum. By locating the node together with the phase jump on top of one of the TA bands, we can assign the observed vibrations either to the ES or to the GS. In our case, the FT of the full oscillatory map reveals the characteristic amplitude node with the corresponding phase jump at 3.7 eV (Figure 3b). The node is located directly on top of the predicted peak of the SE band, while the GS bleaching signals (4.1–4.6 eV) lie outside the probing window. This allows us to unambiguously assign the observed vibrational coherence to the ES.

Moreover, we notice that the spectral position of this phase jump is continuously red-shifting over time (Figure 3c). We attribute this observation to a continuous decrease in the emission energy of the photoexcited state, which is adiabatically followed by the node in the oscillations. Nonequilibrium dynamics modeling the evolution of emission energy due to solvent response confirm that this red-shift is the fingerprint of the L_a_ state (Figures 3d and S9). In contrast, modeling the dynamical response to the solvent of the L_b_ state leads to a negligible change in its emission energy (Figure 3d). These data further confirm that the L_a_ state is populated from early times even in the case of direct excitation of the L_b_ state and support our hypothesis of a hot wave packet coherently exploring both L_b_ and L_a_ regions on the S_1_ surface. The red-shift in the emission energy observed here tracks the solvent-induced changes in the PES topography (described in Figure 2b,c), leading to the stabilization of the L_a_ state below the energy of the L_b_ that facilitates the adiabatic population transfer in 220 fs. The emission shift continues after the energy inversion of the states, while the L_a_ state keeps relaxing and localizing the population in its minimum. This process is associated with the 1 ps time constant found from the global fit (Figure S2b).

We study the nature of the underlying active molecular vibrations by means of a normal-mode analysis (details in the Experimental and Computational Methods section). As it can be conjectured from the nature of the branching plane vectors (Figure 2), high-frequency C–C and C–N stretching modes (>1000 cm^–1^, which are beyond the limits of the temporal resolution of our experimental setup) dominate the ES vibrational dynamics. We identify a mode with 1588 cm^–1^ frequency with a high Huang Rhys (HR) factor only in the L_a_ state (0.50 compared to HR value of 0.00 in L_b_, see Table S3 in the Supporting Information section), which is activated upon vertical excitation. This mode has the highest overlap of all normal modes with the GD vector of the branching plane, which is reflected in the inversion of the L_b_/L_a_ state ordering in a scan along this mode (Figure S6), as such it facilitates the ballistic access to the CI after the initial excitation into the L_a_ state, leading to a L_a_ region on the S_1_ surface after the crossing (by extrapolating the solid red line in Figure 2b onto the S_1_ surface) Below 1000 cm^–1^, we identify a mode with a frequency of 750 cm^–1^ (in good agreement with the experimentally observed 720 cm^–1^ mode), describing distortion of the indole moiety from planarity, which exhibits HR factors of comparable magnitude in both L_b_ (0.34) and L_a_ (0.23). This mode shows the biggest overlap with the DC vector of the branching plane among the excited modes (with significant HR factors) below 1000 cm^–1^, implying strong L_b_/L_a_ wavefunction mixing in the direction associated with the activation of this mode and, together with the aforementioned 1588 cm^–1^ mode, it connects the L_a_ and L_b_ regions on the S_1_ surface by circumventing the CI through the interstate coupling region (red dashed line in Figure 2b connecting the L_a_ and L_b_ regions on S_1_). The observation that the 720 cm^–1^ ES vibrational coherence persists throughout the 1 ps time scale confirms that the branching plane vectors remain active. This observation is also consistent with our interpretation that the wave packet does not remain trapped in the L_b_ valley after the sub-50-fs IC from the L_a_ state but coherently explores the S_1_ surface. This long-lived vibrational coherence is responsible for triggering the solvent relaxation around the L_a_ state, leading to the inversion of the adiabatic state ordering and full repopulation of the L_a_ state. In summary, a theoretical analysis of the active vibrations shows that coherent dynamics along these two modes are immediately initiated upon the photoexcitation of Trp and actively drive the two coherent population transfer events.

## Conclusions

By combining high temporal resolution ultrafast UV transient absorption spectroscopy with the high-level multiconfigurational CASPT2 theory within a hybrid QM/MM setup, we have demonstrated that the primary photoinduced dynamics of tryptophan are coherently driven by the different solvent responses of the vibronically coupled lowest excited states, leading to complex dynamics on the femtosecond time scale.

Initially, the ballistic access to the CI enables sub-50-fs nonadiabatic population transfer from L_a_ to L_b_. Then, the resulting hot wave packet coherently couples the L_a_ and L_b_ regions on the lower adiabatic surface and triggers the solvent response to its polar character by exploring the L_a_ region. Solvent reorganization significantly changes the PES topography and dynamically drives the minimum of the L_a_ state below that of the L_b_ state, promoting the adiabatic transfer of the whole population to the L_a_ state in 220 fs, which is further relaxed in 1.1 ps. This process is supported by a detailed analysis of the oscillations present in the TA map, which shows an excited-state vibrational coherence at 720 cm^–1^ with a characteristic phase jump associated with the red-shifting emission peak that tracks the solvent-induced relaxation of the L_a_ state minimum.

Understanding this highly solvent-sensitive transition can further motivate adopting tryptophan as a probe of the local protein environment even on the ultrafast sub-picosecond time scales, allowing deeper insights into the processes involved in the primary photoinduced protein dynamics.

## Experimental and Computational Methods

Ultrafast TA experiments were carried out using a homemade setup^41^ based on a Ti:sapphire laser generating 100 fs pulses at 800 nm wavelength and 1 kHz repetition rate. Tunable deep UV pump pulses in the 260–290 nm range were generated as second harmonic of a visible noncollinear optical parametric amplifier and compressed to sub-20 fs duration with a prism pair. Probe pulses covering 310–650 nm were obtained through white-light continuum generation by focusing a fraction of the fundamental beam in a calcium fluoride plate. Pump and probe polarizations were set at the magic angle (54.7°). l-Tryptophan (98% purity) was purchased from Sigma-Aldrich and used as received. One hundred eighty-five milligrams of Trp was dissolved in 25 mL of 15 mM ultrapure water-based phosphate buffer solution at pH 7.4, obtaining a concentration of 36.2 mM. The sample was flown in a 150 μm thick laminar liquid jet configuration, resulting in the absorbance of 2 OD at 4.37 eV and 1.9 OD at 4.7 eV pump energy. Steady-state absorption spectra were recorded in a 1 mm fused silica cuvette using a diluted, 4.9 mM solution to avoid the saturation of the spectrophotometer. The used pump fluence was below 300 μJ/cm^2^ to minimize the coherent artifact and solvated electron signals.

Hybrid QM/MM^42^ calculations were executed with the COBRAMM program,^43^ interfacing Gaussian16^44^ and openMOLCAS^45^ QM codes with the AMBER force field. The QM and MM partitioning involved three layers: high, medium, and low. The Trp is treated at the QM level (high layer) and the solvent droplet at the MM level (details in Figure S10). All geometry optimizations were done while allowing the nearest two solvent shells to relax to the QM solute (medium layer), while the rest of the MM water molecules were frozen (low layer). The ground-state geometry minimum was obtained at the Møller–Plesset second-order perturbation theory (MP2) level. The excited-state geometry optimizations were computed at the SS-CASPT2 level using an active space of 10 electrons in nine orbitals (0|10,9|0) including all of the valence π-orbitals. All computations utilized the ANO-L-VDZP basis set^46^ employing the Cholesky decomposition method to speed up the computation of atomic integrals. All CASPT2^47^ computations were done with zero IPEA shift and an imaginary shift^48^ of 0.2. The simulation of linear absorption spectra was done in a displaced harmonic oscillator formalism with the gradients and energies computed at the XMS-CASPT2^49^ level with an augmented active space of (0,0|10,9|2,4) with four extra virtual orbitals in the RAS3 subspace. The computation was performed with the program FCClasses3 utilizing the vertical gradient (VG) approximation and incorporating temperature effects.^50−52^ PA energies and oscillator strengths for the simulation of transient spectra were calculated at the SS-CASPT2 level, state averaging over 20 singlet states in the underlying CASSCF wavefunction. The PA computations displayed in Figure 1e were done by augmenting the CASSCF space with four additional orbitals in the RAS3 subspace (0,0|10,9|2,4) to have the required flexibility in the description of high-energy excited states accessed during ultrafast TA experiments. The theoretical spectra were obtained by broadening the CASPT2 vertical excitations with Gaussians with an empirical width of 0.15 eV.

The branching space displayed in Figure 2 is computed at RMS-CASPT2,^53^ which includes the interstate coupling elements in the Hamiltonian required for the correct description of the topography in the vicinity of a CI, in contrast to state-specific SS-CASPT2. A detailed discussion of the branching space computed with various flavors of CASPT2 (SS, MS, XMS) justifying the use of RMSPT2 surface is included in Supporting Information, Section 11.

Nonequilibrium dynamics were carried out in a fully molecular mechanics scheme with AMBER^54^ by inserting Merz–Kollman charges^55^ fitted to CASPT2 density using Multiwfn.^56,57^ The dynamics were initialized on 100 decorrelated solvent snapshots obtained by solvent sampling around restrained solute. During the nonequilibrium dynamics, the solute was restrained by harmonic forces. The electronic structure computations along the dynamics for modeling transient spectra (Figure S7) and computing emission energies (Figure 3d) were done at the SS-CASPT2 level. Before computing the SS-CASPT2 energies on selected snapshots from dynamics, the respective SS-CASPT2-optimized geometries of L_a_ and L_b_ minimum were re-inserted in place of the MM solute. To avoid any kind of solvent bias, 100 trajectories were run for 5000 fs and the reported values were computed taking the ensemble average over the 100 copies.

## Supporting Information Available

TA maps for different pump photon energies; global fit of the experimental TA data; calculated Trp^+^ cation spectra; and L_a_ and L_b_ charge distributions (PDF)

## Abbreviations

GS: ground state
ES: excited state
IC: internal conversion
FC: Franck–Condon
CI: conical intersection
TA: transient absorption
PES: potential energy surface
PA: photoinduced absorption
Se: stimulated emission
QM: quantum mechanics
MM: molecular mechanics
CASSCF: complete active space self-consistent field
CASPT2: complete active space perturbation theory 2
