# Supplementary material for: Environment-Driven Coherent Population Transfer Governs the Ultrafast Photophysics of Tryptophan
Source: J Am Chem Soc. 2022 Jul 7;144(28):12884–92. doi: 10.1021/jacs.2c04565 (PMC9305959; doi:10.1021/jacs.2c04565)
Supplement: Supplementary file 1 — ja2c04565_si_001.pdf [file ja2c04565_si_001.pdf]

Supporting Information

Environment driven coherent population transfer governs the ultrafast photophysics of tryptophan

Vishal Kumar Jaiswal[1], Piotr Kabaciński[2], Barbara E. Nogueira de Faria[3], Marziogiuseppe Gentile[1], Ana Maria de Paula[3], Rocio Borrego-Varillas[4], Artur Nenov[1], Irene Conti[1], Giulio Cerullo[2,4], Marco Garavelli[1]

[1] Dipartimento di Chimica industriale "Toso Montanari", Università di Bologna, Viale del Risorgimento 4, 40136 Bologna, Italy;

[2] Dipartimento di Fisica, Politecnico di Milano, Piazza Leonardo da Vinci 32, 20133 Milano, Italy;

[3] Departamento de Física, Universidade Federal de Minas Gerais, 31270-901 Belo Horizonte-MG, Brazil;

[4] Istituto di Fotonica e Nanotecnologie, CNR-IFN, Piazza Leonardo da Vinci 32, 20133 Milano, Italy

1. Transient absorption maps for different pump photon energies.

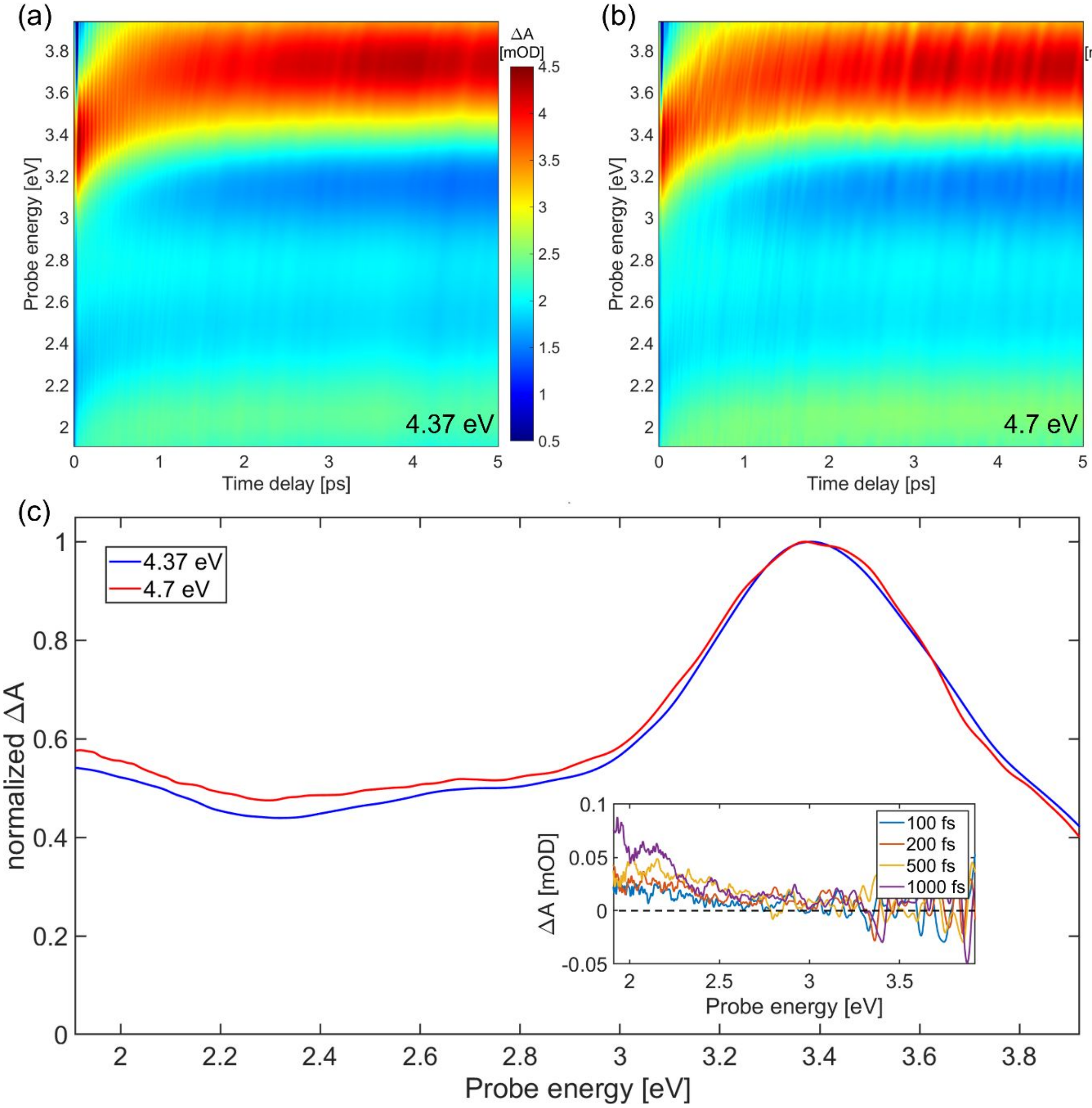

Figure S1: Comparison of transient absorption (TA) maps for Trp excited with (a) 4.37 eV and (b) 4.7 eV pump pulse. The pump pulse is tuned to excite predominantly either the L_a or the L_b electronic state, to evaluate the differences in the decay pathways associated with each initial state. The maps show remarkable similarity, suggesting that the photoinduced dynamics observed after 50 fs from excitation are independent of the nature of the initially populated state. (c) Normalized TA spectra at 100 fs delay for the two excitation energies used, showing more intense signal in the range where solvated electron signal peaks for the higher energy pump that leads to increased photoionization yield. TA of solvent pumped at 4.37 eV is shown as an inset with signals characteristic of solvated electron absorption, but of very low intensity. We note that when tryptophan is present in the solution, it absorbs most of the pump photons, further decreasing solvated electrons created from ionization of water, thus the solvated electron signal observed in the Trp TA maps is only originating from Trp ionization. The high level of noise is caused by only a few TA scans done for this measurement.

2. Global fit of the experimental TA data.

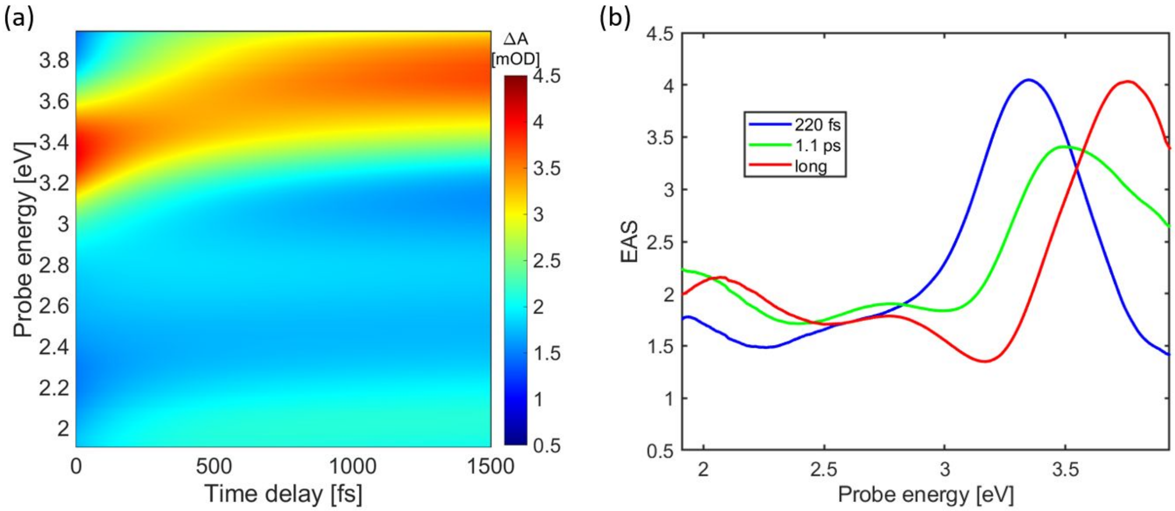

Figure S2: (a) TA map reconstructed based on the global fit and (b) corresponding evolution associated spectra (EAS) revealing the two time constants of 220 fs and 1.1 ps that describe the disappearance of the strong PA band at 3.37 eV (PA_1) and the simultaneous appearance of a band at 3.76 eV (PA_2) on the picosecond timescale, and the remaining long lived spectrum of the relaxed L_a state. The global fitting procedure has been done using Glotaran software [1].

3. Calculated L_a, L_b and Trp^+ cation spectra.

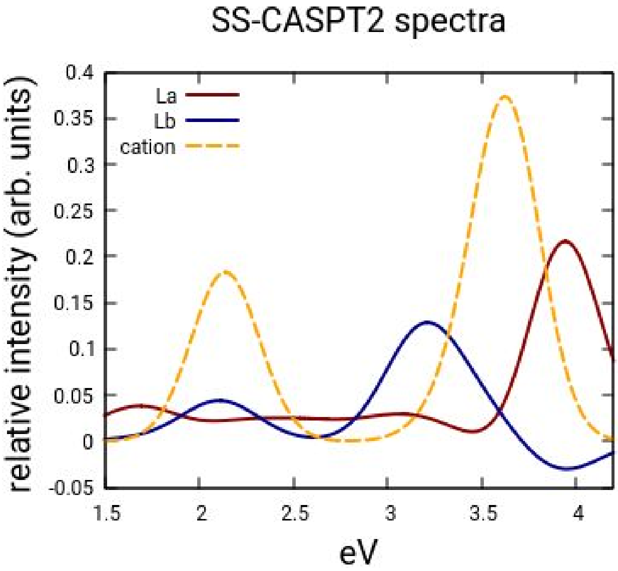

Figure S3: TA spectra, calculated at the SS-CASPT2 level, of the L_a state, L_b state and Trp^+ cation (dashed yellow line), showing two peaks around 2 eV and 3.5 eV.

Table S1. QM/MM SS-CASPT2 theoretical TA energies (eV) and magnitude of transition dipole moment squared ($|TDM|^2$) of L_b, L_a and Trp^+ cation. Negative energetic values are used to denote emission. Estimations from the corresponding optimized ES minima

L_b-ESA		L_a ESA		Trp^+ absorption	
**Energy (eV)**	**$\|TDM\|^2$**	**Energy (eV)**	**$\|TDM\|^2$**	**Energy (eV)**	**$\|TDM\|^2$**
-3.92	0.249952	-3.81	0.88024	1.03	0.015673
0.5	0.208048	0.16	1.051743	2.14	0.618173
1.66	0.021464	1.53	0.250453	3.34	0.066337
1.71	0.016875	1.74	0.416498	3.63	0.725782
2.22	0.004615	2.27	0.057867	4.42	0.036306
1.89	0.013195	2.2	0.155162	4.85	0.159675
2.11	0.525601	2.59	0.163152	4.67	1.004769
3.27	0.051236	2.45	0.028579	5.12	0.245154
2.34	0.010322	2.97	0.15512	5.14	0.119069
3.48	0.229422	3.22	0.140665		
3.18	0.914638	3.91	0.08543		
3.9	0.033001	3.9	0.719787		
4.34	0.026716	3.9	1.38115		

4. L_a and L_b Charge Distribution

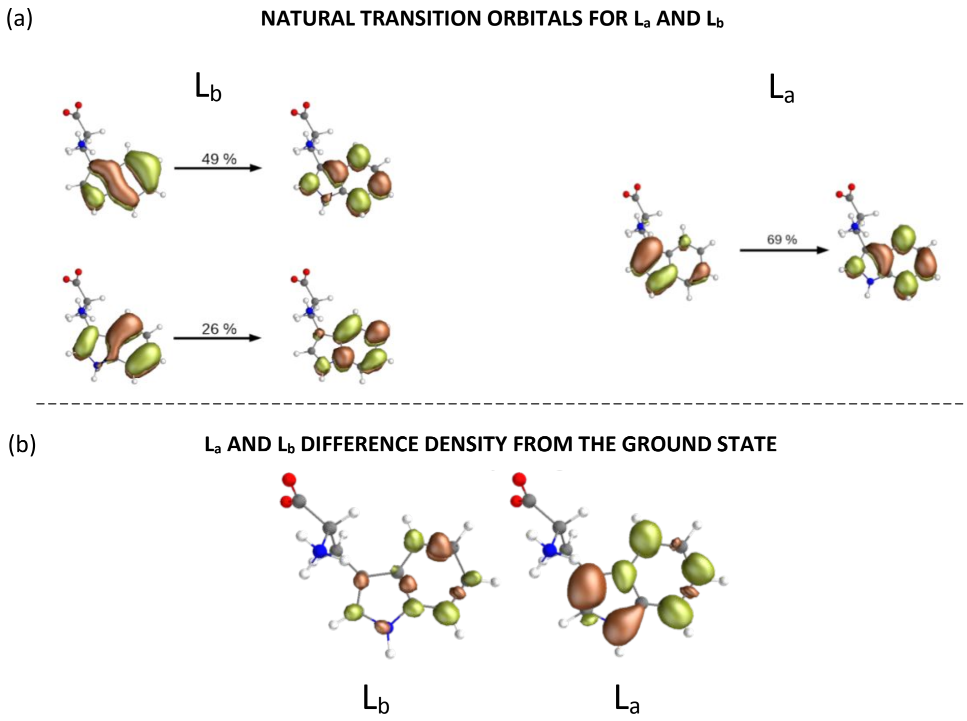

Figure S4. (a) Natural transition orbitals for the brightest $\pi\pi^*$ state (L_b) and for $\pi\pi^*$ L_a state at Franck-Condon region; (b) Difference electron density for L_b and L_a with respect to the ground state. Red and green signify regions of electron depletion and addition compared to ground state electron density, respectively.

5. Time Evolution of L_a and L_b Energy Gap.

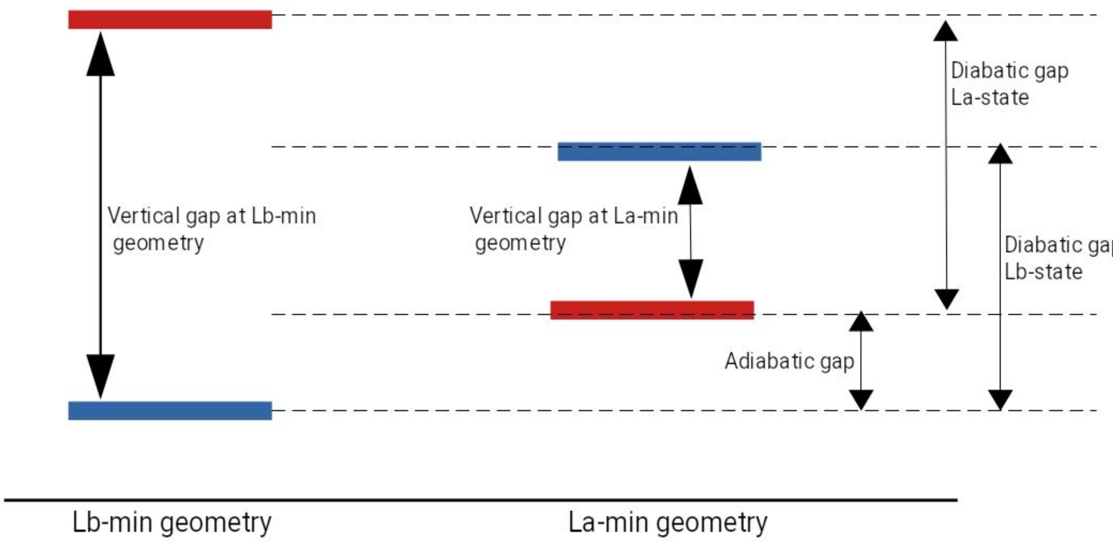

Figure S5. The various adiabatic and diabatic gaps at critical geometries. See Table S2 for the time evolution of these gaps at SS-CASPT2 level.

Table S2: Time-evolution of various gaps (see Figure S5) in non-equilibrium dynamics around L_a-state computed at SS-CASPT2 level with |10,9| active-space. The values shows are average of 100 structures on which the dynamics were performed.

	Vertical Gap L_a-min	Vertical Gap L_b-min	Adiabatic gap S_1-min	Diabatic gap L_b	Diabatic gap L_a
FC	-0.110	0.406	0.098	0.210	-0.307
80fs	-0.271	0.211	-0.105	0.166	-0.316
200fs	-0.273	0.189	-0.101	0.173	-0.289
520fs	-0.337	0.141	-0.219	0.118	-0.407
1000fs	-0.332	0.125	-0.306	0.016	-0.478

6. Branching Space: Excited modes responsible for non-adiabatic population transfer

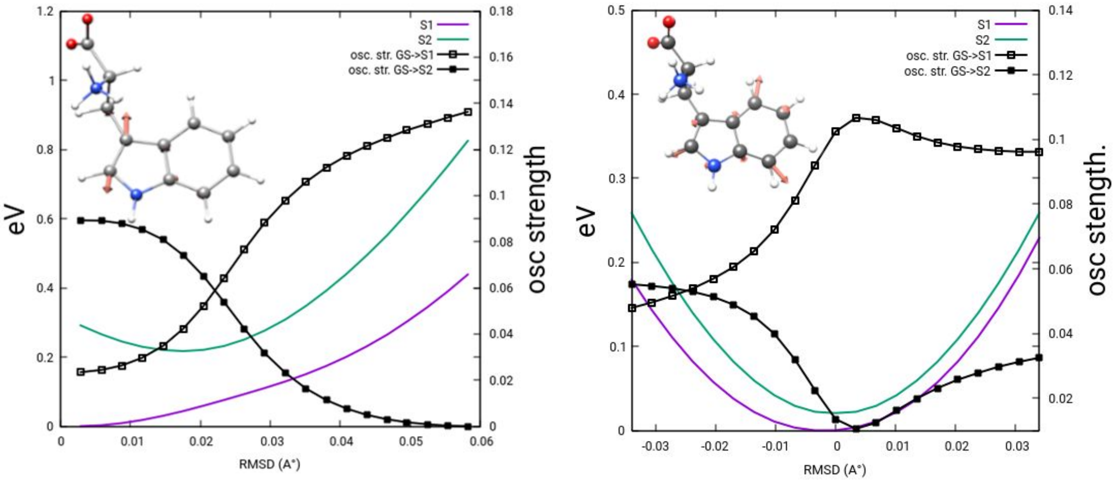

Figure S6. Scan along the two major modes activated upon vertical excitation from Franck-Condon (FC) geometry. The energies (in eV on left y-axis/ colored lines) of the adiabatic S1/S2 surfaces are shown along with oscillator strength (relative intensity on right y-axis / black linepoints) for the transition from the ground-state. These two modes have significant overlaps with the branching plane vectors and are responsible for non-adiabatic population transfer through vibronic coupling of L_a/L_b electronic states. (a) Scan along 1588 cm^{-1} mode from FC geometry. This mode has high overlap with the gradient difference (GD) vector of L_a/L_b states. The scan leads to an inversion of L_a/L_b through weakly avoided crossing. (b) Scan along 752 cm^{-1} mode around the conical intersection. This mode has high overlap with the derivative coupling (DC) vector. The scan around conical intersection leads to regions of L_a-L_b mixing.

7. L_a transient signals along the solvent reorganization dynamics.

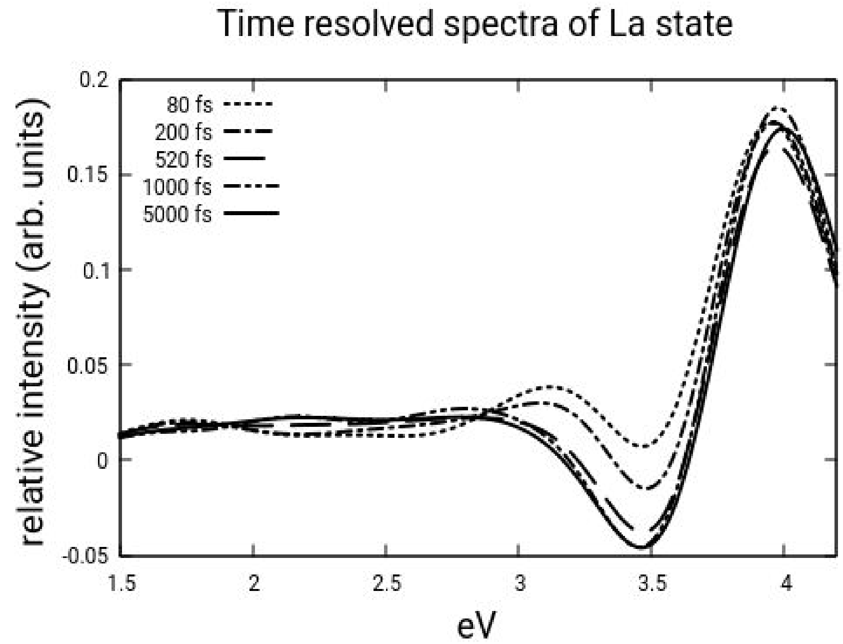

Figure S7. The time resolved spectra of L_a-state was computed over the structures from non-equilibrium dynamics. Any spectra at a particular time was the average of computations over 100 snapshots. The computations were done at SS-CASPT2 level.

8. Fourier transform maps of the oscillatory component of the signal with two different pump photon energies.

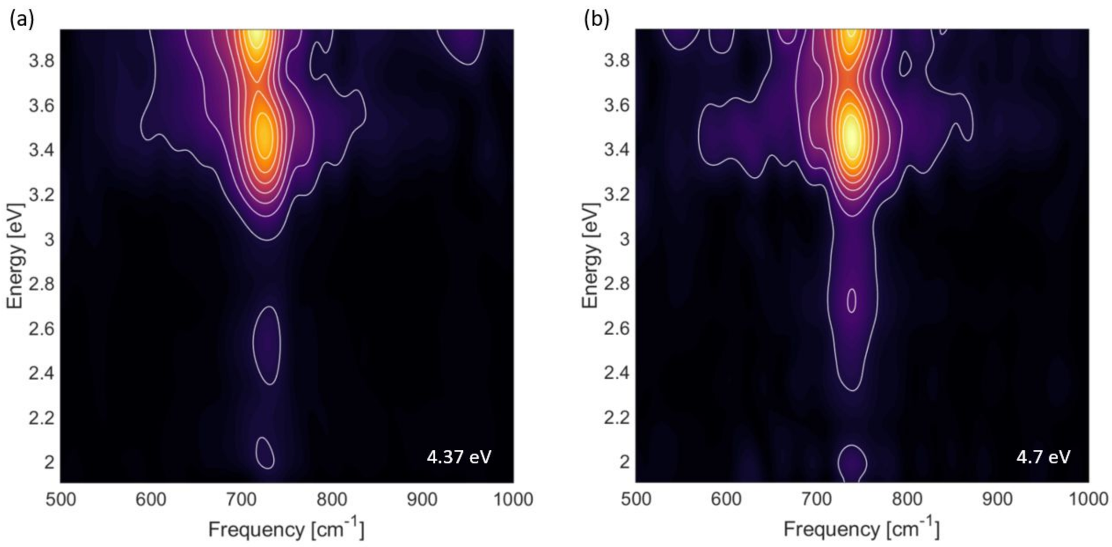

Figure S8: Comparison of FT maps for Trp excited with (a) 4.37 eV and (b) 4.7 eV pump pulse. While the differential absorption signal shown in Fig. S1 was identical for the two pump photon energies, the oscillatory component has clear differences. The main peaks are located at 720 cm^{-1} for the 4.37 eV pump and at 740 cm^{-1} for the 4.7 eV pump. In addition to that, some weaker peaks are more or less pronounced between the measurements, and their position in the probe spectrum varies as well in the visible region. For example, the peak at 2.5 eV in panel (a) is still present in panel (b) but now there is also a stronger peak at 2.7 eV overlapping with it. This result shows that the vibrational coherence observed here preserves the memory of which state has been initially excited.

9. Phase jump shift for two different pump energies overlapped with the theoretical L_a emission

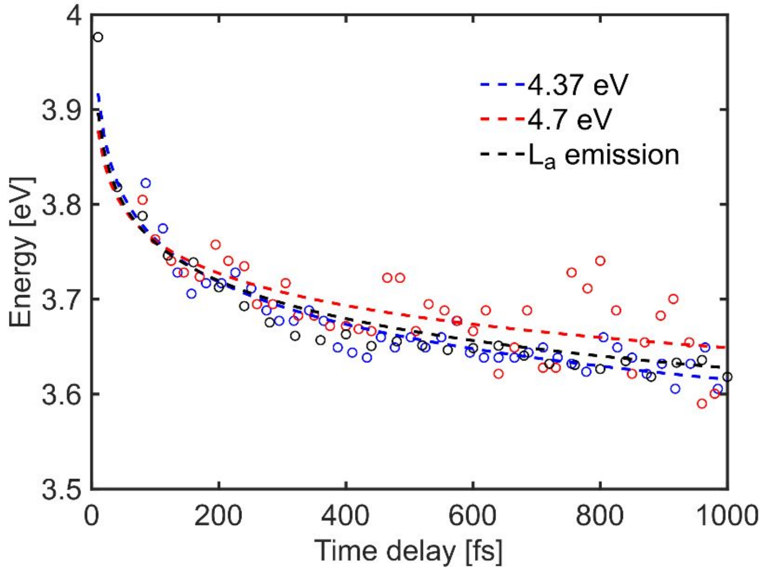

Figure S9: Comparison of phase jump shift for the two different pump photon energies with the theoretical L_a emission energy. Theoretical data has been uniformly shifted by 0.11 eV to facilitate comparison.

Table S3. L_a and L_b Huang-Rhys Factors for each mode.

MP2 Frequencies	Lb	La	MP2 Frequencies	Lb	La	MP2 Frequencies	Lb	La	MP2 Frequencies	Lb	La
**30.2616**	0.0034	0.0099	**675.454**	0.0017	0.004	**1146.095**	0.0015	0.0018	**1588.754**	0.0004	0.5025
**87.1706**	0.003	0.1776	**708.6298**	0.0246	0.0057	**1159.043**	0.0401	0.0737	**1616.213**	0.0018	0.0502
**137.4788**	0.233	0.5038	**718.1794**	0.02	0.0522	**1163.791**	0	0.0435	**1649.137**	0.0001	0.0269
**187.0386**	0.2665	0.0156	**745.1002**	0.0135	0.0413	**1200.115**	0.0029	0.0525	**1660.134**	0.0006	0.0417
**197.9587**	0.0062	0.057	**752.5991**	0.3409	0.2349	**1242.339**	0.0492	0.035	**1698.232**	0.0001	0
**218.1998**	0.0543	0.5264	**775.294**	0.0448	0.0857	**1255.864**	0	0.0728	**1712.651**	0.0002	0.003
**226.0516**	0.0306	0.0037	**802.119**	0.068	0.2762	**1279.528**	0.0996	0.0206	**3085.927**	0.0001	0
**233.3158**	0.0059	0.0953	**817.6869**	0.0001	0.033	**1307.622**	0.0008	0.0036	**3139.175**	0	0
**257.5746**	0.0017	0.0015	**838.0504**	0.0134	0.007	**1329.292**	0.0208	0.0297	**3172.982**	0.0002	0.0008
**339.0144**	0.0058	0.0006	**871.3543**	0.0009	0.1153	**1384.9**	0.0013	0.0285	**3200.005**	0.0001	0.0001
**358.4333**	0.0089	0.0308	**889.4647**	0.0007	0.0016	**1396.21**	0.0027	0.1258	**3224.034**	0	0.0001
**409.7918**	0.0147	0.0002	**924.2294**	0.0028	0.0116	**1399.13**	0.0223	0.0272	**3232.432**	0.0006	0.0036
**426.4277**	0.0014	0.0014	**933.6901**	0.0041	0.0101	**1456.781**	0.0142	0.005	**3273.758**	0.0001	0.0008
**471.9441**	0.0108	0.0001	**943.974**	0.0101	0.0973	**1459.54**	0.0282	0.018	**3292.554**	0.0003	0.0005
**514.6048**	0.0025	0.022	**989.6248**	0.0032	0.0062	**1466.95**	0.0025	0.0081	**3299.129**	0.0027	0.0021
**540.9589**	0.015	0.0117	**1024.383**	0.1737	0.0283	**1486.465**	0.0497	0.0005	**3432.941**	0	0.0009
**564.246**	0.0198	0.0027	**1062.677**	0.0037	0.0208	**1511.295**	0.0361	0.0003	**3482.121**	0.0001	0
**578.8857**	0.1125	0.1221	**1097.252**	0.008	0.0097	**1533.656**	0.0368	0.1623	**3510.925**	0.0011	0.0007
**591.0905**	0.0129	0.0036	**1125.912**	0.0116	0.014	**1549.332**	0.026	0.0329			

10. QM/MM Setup.

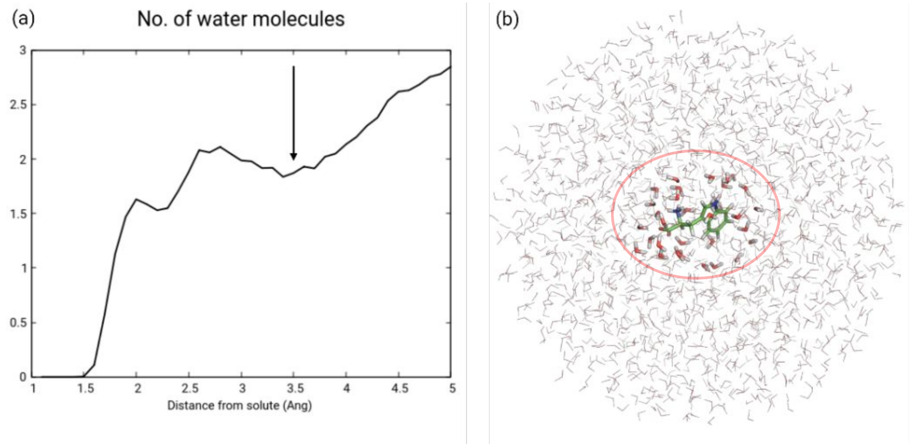

Figure S10. QM/MM setup showing High/Medium/Low (HML) partitioning. High(QM)//Medium(movable MM)//Low(fixed MM). This setup is utilized for optimizations, computations of MEP and computation of transient spectra. The MM movable Medium layer, includes the nearest two solvent shells of water molecules to the QM solute, shown inside the red circle (panel (b)). Panel (a) shows that the second shell of waters around the Trp lies in a distance of around 3.5 Å from the molecule. The entire water droplet radius is 20 Å.

11. Topology of the adiabatic surfaces at different CASPT2 levels.

Panel (a) of Figure S12 shows the topology of the adiabatic surfaces across the branching plane coordinates with initial solvent distribution at various levels of CASPT2. The colour plot on xy-plane is the transition dipole moment of the transition to lower adiabatic surface from ground-state. The colour coded TDM helps to characterize the regions of L_a and L_b on the lower surface. The computations have been done at single-state CASPT2(SS-PT2) which is just correction of CASSCF energies at PT2 level, and various flavors of multistate PT2 (MS, XMS and RMS) where the CASSCF states are rotated to diagonalize the PT2 Hamiltonian. The reference computation is done at XMS-CASPT2 with an active-space of 10 electrons in 9 orbitals in RAS2 space augmented with 4 extra virtual orbitals in RAS3 space, referred as aug-XMSPT2. All the other (SS,MS,RMS and XMS) computations are done with active-space of 10 electrons in 9 orbitals.

The topology with an expanded active-space 0|10,9|2,4 at XMS level(also referred as aug-XMSPT2) is considered as the reference when assessing the other surfaces computed at the smaller |10,9| active-space. With this consideration, the topology at RMS level with the smaller |10,9| active-space looks most similar to the reference showing a stabilization of L_b region compared to L_a-region. Curiously the topology of XMS at |10,9| active-space shows the opposite tendency to the reference with a slight stabilization in L_a-region. Single-state CASPT2 (SSPT2) does not describe the branching plane but locates a conical intersection seam instead, and the electronic character of the surfaces show little L_a/L_b mixing across the plane. It correctly describes the stabilization of L_b region versus L_a region in the Franck-Condon distribution of solvent. In contrast MS-CASPT2 has the most different topology compared to all the other methods showing a flat long valley connecting L_a and L_b regions along GD vector.

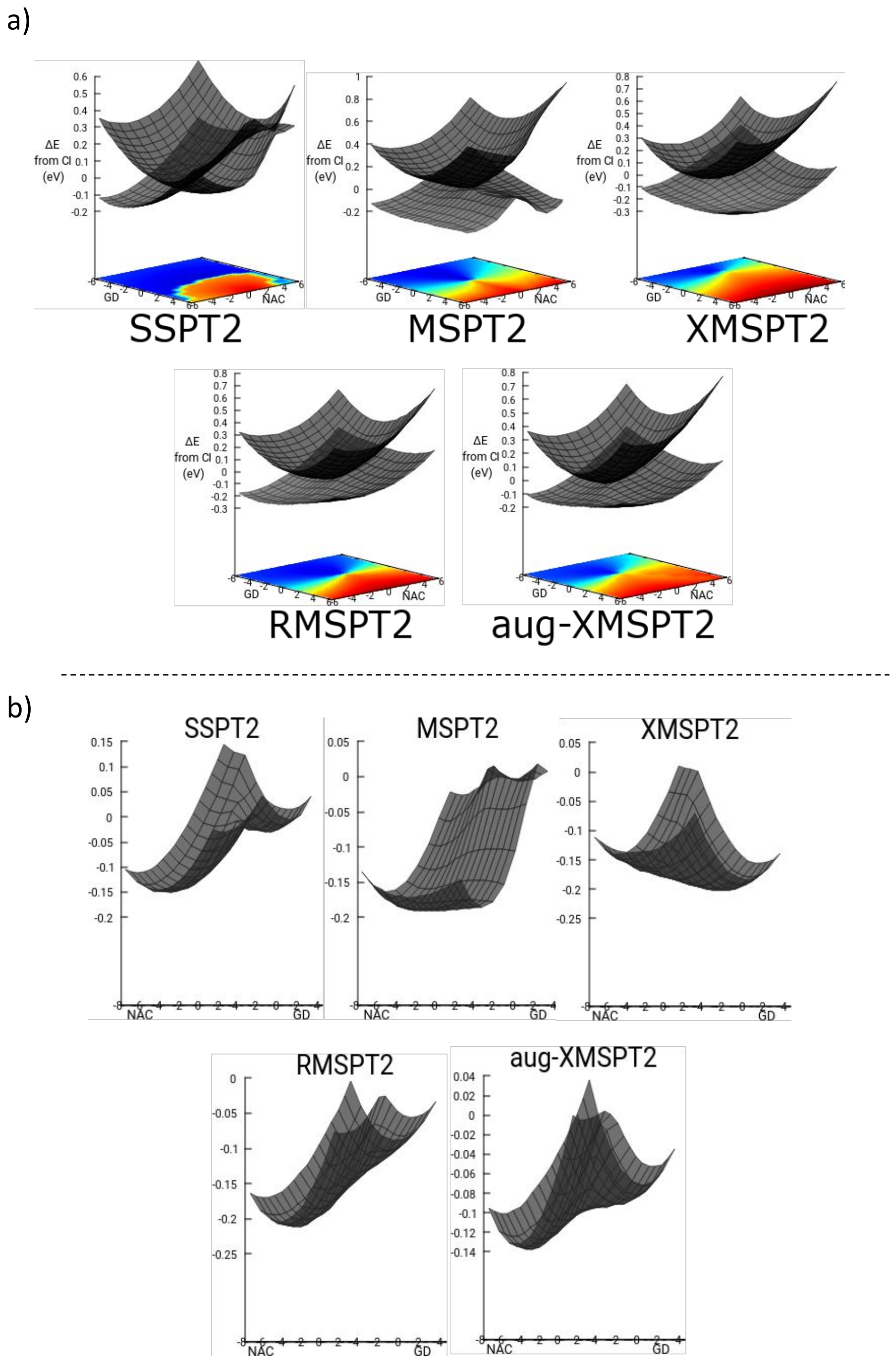

Figure S11. (a) The topology of the adiabatic surfaces across the branching plane coordinates with initial solvent distribution at various levels of CASPT2 calculations. (b) A side-view of the same surfaces as the above figure to enhance the L_a vs L_b stabilization on the S_1 surface across various levels of PT2 theory.

References:

1. Snellenburg JJ, Laptenok SP, Seger R, Mullen KM, van Stokkum IHM (2012). “Glotaran: A Java-Based Graphical User Interface for the R Package TIMP.” Journal of Statistical Software, 49(3), 1–22.
